# Ectopic thyroid in an adrenal mass: a case report

**DOI:** 10.1186/1471-2490-6-18

**Published:** 2006-08-10

**Authors:** Jun Hagiuda, Isao Kuroda, Takuji Tsukamoto, Munehisa Ueno, Chizuko Yokota, Takanori Hirose, Nobuhiro Deguchi

**Affiliations:** 1 Department of Urology, Saitama Medical University, Saitama, Japan; 2 Department of Internal Medicine, Saitama Medical University, Saitama, Japan; 3 Department of Pathology, Saitama Medical University, Saitama, Japan; 4 Department of Urology, Saitama Medical University, 38 Morohongo. Moroyamamachi, Iruma, Saitama 3500495, Japan

## Abstract

**Background:**

It is difficult to explain ectopic thyroid beneath the diaphragm because during the development the thyroid descends from the tongue to the anterior of the trachea. A few cases of ectopic lesions have been reported in the literature for abdominal organs including the adrenal glands, but the mechanism by which the thyroid components migrate into the abdomen has been poorly understood.

**Case presentation:**

A 54-year-old woman was diagnosed as having an adrenal mass. Laparoscopic adrenalectomy was carried out. Microscopically, the mass was composed of normal adrenal and ectopic thyroid tissues.

**Conclusion:**

We herein describe the fourth case reported of ectopic thyroid in the adrenal gland.

## Background

The cause of ectopic thyroid in the adrenal glands is unknown. The development of the thyroglossal duct can cause an undescended thyroid anywhere between the base of tongue and the normal thyroid position. However, the failure of migration of thyroid tissue has been reported to produce ectopic thyroid even in subdiaphragmatic organs such as the gallbladder [1], the mesentery of the small intestine [2], and the adrenal glands [3,4].

In the present paper, we report our experience with a patient with an adrenal tumor that was shown to be composed of thyroid tissues, and we review similar cases in the literature.

## Case presentation

**Figure 1:**
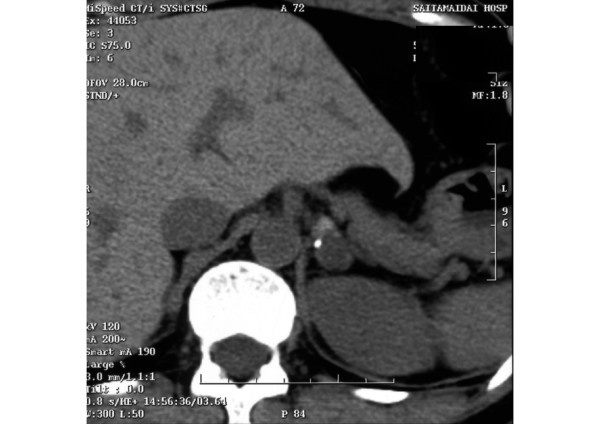
CT shows a 16 × 10 mm left adrenal mass.

**Figure 2:**
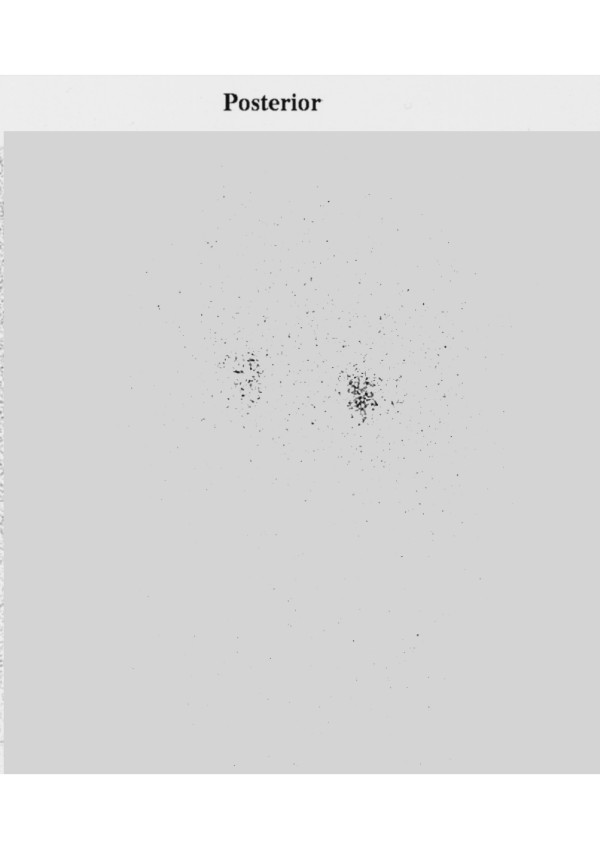
Adrenal scintigraphy reveals the uptake of I^131^-adosterol by the left adrenal gland.

**Figure 3:**
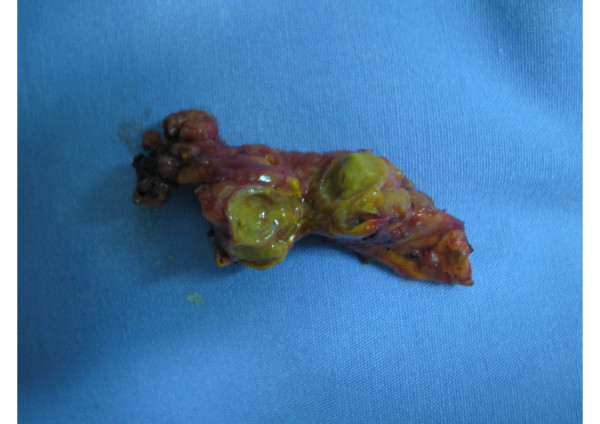
Surgical specimen of the adrenal gland contains an 8 mm cystic mass.

**Figure 4:**
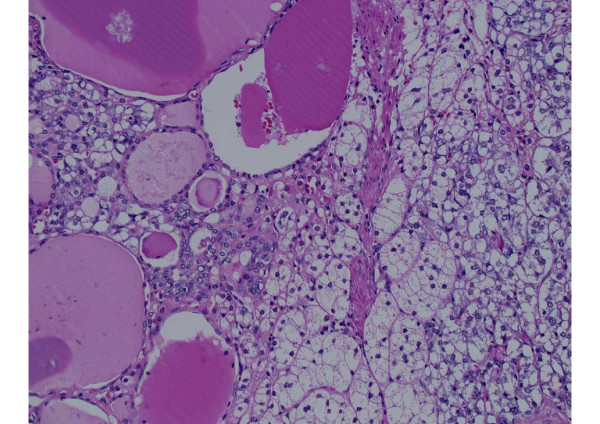
Photomicrograph of thyroid tissue in the adrenal gland. H&E section shows that the follicle contained eosinophilic colloid-like material lined by a single-layered epithelium (arrow) (×400).

**Figure 5:**
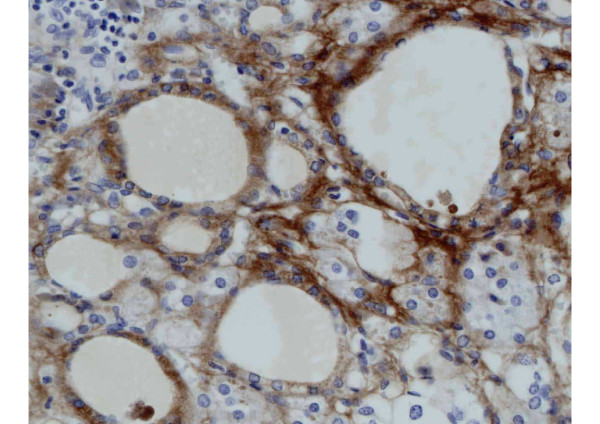
Thyroglobulin immunohistochemical stain and.

**Figure 6:**
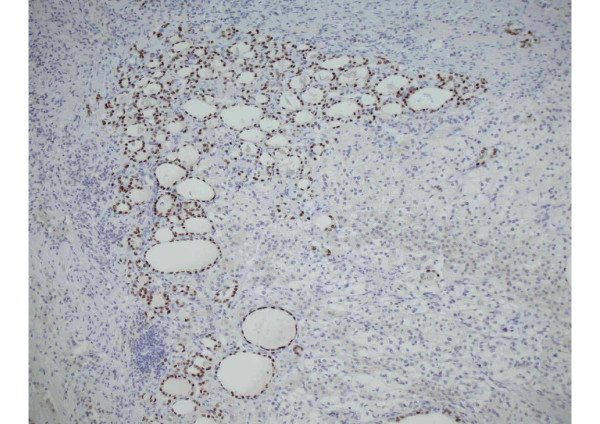
TTF-1 stain is positive for the epithelial cells.

A 54-year-old woman was admitted to our hospital for evaluation of hypertension. Routine laboratory study showed hypokalemia (K 3.3 mEq/l). Endocrine studies including T3, T4, TSH, adrenocorticotropic hormone, and renin showed normal. The aldosterone level was elevated to 195 pg/ml (normal range: 30–159 pg/ml). Computed tomography (CT) detected a low density left adrenal mass 16 × 12 mm with small calcification Fig. 1. Adrenal scintigraphy revealed the uptake of I^131^-adosterol by the left adrenal gland (Fig. 2). Because these diagnostic methods taken together could not rule out primary aldosteronism or malignant tumors, the patient underwent laparoscopic adrenalectomy. The surgical specimen was a 40 × 20 mm adrenal gland tissue containing an 8 mm cystic mass (Fig. 3). Histological examination revealed an admixture of adrenal gland and thyroid tissue composed of many follicles lined by a single-layered epithelium. Each follicle contained an eosinophilic colloid-like material (Fig. 4). The epithelial cells in the follicles showed immunoreactivity for cytokeratin, thyroglobulin (Fig. 5), and thyroid transcription factor-1 (TTF-1) (Fig. 6).

After the surgery, imaging examinations using MRI and I^123^-scaning thyroid scintigraphy failed to show any lesions in the thyroid. No evidence of other tumors was detected by a whole body CT scan and ultrasound. The patient's postoperative course was uneventful, and serum TSH, free T3, and free T4 levels were normal. Although this patient has been followed up for 2 years after the operation, laboratory study and computed tomography showed no any lesions.

## Conclusion

The thyroid tissue starts developing during the fourth embryonic week. It appears on the tongue as an epithelial growth. By the seventh embryonic week, the thyroid gland descends to the adult position, anterior to the trachea. Ectopic thyroid gland can be found along this pathway, like the region of the neck, the mediastinum, the pharynx, the larynx, the esophagus, the trachea, and around the aorta. On the other hand, intra-abdominal thyroid tissues have been reported scattered within the gallbladder [1], the mesentery of the small intestine [2], the pancreas [5], the porta hepatitis [6], the duodenum [7], and the space posterior to the spleen and stomach [8].

Some explanations have been suggested for ectopic thyroid. First, metaplasia in nature or that it represents either choristomatous tissue or a teratoma [7] cannot be excluded. Our case cannot be explained in terms of a metaplastic transformation because the thyroid develops from the endoderm, whereas the adrenal gland develops from the ectoderm and mesoderm. No evidence of teratomatous elements was found. Secondly, all papers reported that ectopic thyroid tissues were limited to the upper abdomen. This suggests that the present case is possibly caused by over-descending hypoglossal duct remnants as well as other manifestations of ectopic thyroid beneath the diaphragm.

Three cases have been reported of ectopic thyroid tissue found in the adrenal gland [3,4]. Tsujimura et *al*. described the first case, in which a 61-year-old-woman incidentally presented with a cystic lesion in the right adrenal gland. The patient was diagnosed as having ectopic thyroid tissues because the resected tumor was composed of large follicles and follicular epithelium in the cyst wall that stained positively for thyroglobulin. The clinical and pathological features of the two other cases were mostly identical to those of the first reported case and the present case. Because of the recent development of routine health checks, it is now expected that the finding of an adrenal mass with a cystic lesion will occur in an incidental manner. Some diseases, like cortical adenoma, adenocarcinoma, pheochromocytoma, and dermoid cyst are reported to form adrenal cysts [9]. Ectopic thyroid is one possibility of a differential diagnosis when it shows both a cystic lesion and normal hormonal data.

## Competing interests

The author(s) declare that they have no competing interests.

## Authors' contributions

JH designed this case report and drafted the manuscript. IK, TT and CY carried out the operation of this patient and helped to draft the manuscript. MU and ND participated in the design of the study and helped to draft the manuscript. TH carried out the pathological examination. All authors read and approved the final manuscript.

## Pre-publication history

The pre-publication history for this paper can be accessed here:
